# GMMID: genetically modified mice information database

**DOI:** 10.1093/database/baae078

**Published:** 2024-08-20

**Authors:** Menglin Xu, Minghui Fang, Qiyang Chen, Wenjun Xiao, Zhixuan Xu, Bao Cai, Zhenyang Zhao, Tao Wang, Zhu Zhu, Yingshan Chen, Yue Zhu, Mingzhou Dai, Tiancheng Jiang, Xinyi Li, Siuwing Chun, Runhua Zhou, Yafei Li, Yueyue Gou, Jingjing He, Lin Luo, Linlin You, Xuan Jiang

**Affiliations:** Shenzhen Key Laboratory for Systems Medicine in Inflammatory Diseases, School of Medicine, Shenzhen Campus of Sun Yat-Sen University, 66#, Gongchang Road, Shenzhen, Guangdong 518107, China; School of Intelligent Systems Engineering, Sun Yat-Sen University, 66#, Gongchang Road, Shenzhen, Guangdong 518107, China; Guangdong Key Laboratory of Intelligent Transportation Systems (ITS), Sun Yat-Sen University, 66#, Gongchang Road, Shenzhen, Guangdong 518107, China; School of Intelligent Systems Engineering, Sun Yat-Sen University, 66#, Gongchang Road, Shenzhen, Guangdong 518107, China; Guangdong Key Laboratory of Intelligent Transportation Systems (ITS), Sun Yat-Sen University, 66#, Gongchang Road, Shenzhen, Guangdong 518107, China; Shenzhen Key Laboratory for Systems Medicine in Inflammatory Diseases, School of Medicine, Shenzhen Campus of Sun Yat-Sen University, 66#, Gongchang Road, Shenzhen, Guangdong 518107, China; Shenzhen Key Laboratory for Systems Medicine in Inflammatory Diseases, School of Medicine, Shenzhen Campus of Sun Yat-Sen University, 66#, Gongchang Road, Shenzhen, Guangdong 518107, China; Shenzhen Key Laboratory for Systems Medicine in Inflammatory Diseases, School of Medicine, Shenzhen Campus of Sun Yat-Sen University, 66#, Gongchang Road, Shenzhen, Guangdong 518107, China; Shenzhen Key Laboratory for Systems Medicine in Inflammatory Diseases, School of Medicine, Shenzhen Campus of Sun Yat-Sen University, 66#, Gongchang Road, Shenzhen, Guangdong 518107, China; Guangdong GemPharmatech Co,Ltd, No 6, Qianjin West Rd. Shishan Town Nanhai District, Foshan, Guangdong 528225, China; Guangdong GemPharmatech Co,Ltd, No 6, Qianjin West Rd. Shishan Town Nanhai District, Foshan, Guangdong 528225, China; Guangdong GemPharmatech Co,Ltd, No 6, Qianjin West Rd. Shishan Town Nanhai District, Foshan, Guangdong 528225, China; Shenzhen Key Laboratory for Systems Medicine in Inflammatory Diseases, School of Medicine, Shenzhen Campus of Sun Yat-Sen University, 66#, Gongchang Road, Shenzhen, Guangdong 518107, China; Shenzhen Key Laboratory for Systems Medicine in Inflammatory Diseases, School of Medicine, Shenzhen Campus of Sun Yat-Sen University, 66#, Gongchang Road, Shenzhen, Guangdong 518107, China; Shenzhen Key Laboratory for Systems Medicine in Inflammatory Diseases, School of Medicine, Shenzhen Campus of Sun Yat-Sen University, 66#, Gongchang Road, Shenzhen, Guangdong 518107, China; Shenzhen Key Laboratory for Systems Medicine in Inflammatory Diseases, School of Medicine, Shenzhen Campus of Sun Yat-Sen University, 66#, Gongchang Road, Shenzhen, Guangdong 518107, China; Shenzhen Key Laboratory for Systems Medicine in Inflammatory Diseases, School of Medicine, Shenzhen Campus of Sun Yat-Sen University, 66#, Gongchang Road, Shenzhen, Guangdong 518107, China; Shenzhen Key Laboratory for Systems Medicine in Inflammatory Diseases, School of Medicine, Shenzhen Campus of Sun Yat-Sen University, 66#, Gongchang Road, Shenzhen, Guangdong 518107, China; Shenzhen Key Laboratory for Systems Medicine in Inflammatory Diseases, School of Medicine, Shenzhen Campus of Sun Yat-Sen University, 66#, Gongchang Road, Shenzhen, Guangdong 518107, China; Shenzhen Key Laboratory for Systems Medicine in Inflammatory Diseases, School of Medicine, Shenzhen Campus of Sun Yat-Sen University, 66#, Gongchang Road, Shenzhen, Guangdong 518107, China; Shenzhen Key Laboratory for Systems Medicine in Inflammatory Diseases, School of Medicine, Shenzhen Campus of Sun Yat-Sen University, 66#, Gongchang Road, Shenzhen, Guangdong 518107, China; Shenzhen Key Laboratory for Systems Medicine in Inflammatory Diseases, School of Medicine, Shenzhen Campus of Sun Yat-Sen University, 66#, Gongchang Road, Shenzhen, Guangdong 518107, China; School of Intelligent Systems Engineering, Sun Yat-Sen University, 66#, Gongchang Road, Shenzhen, Guangdong 518107, China; Guangdong Key Laboratory of Intelligent Transportation Systems (ITS), Sun Yat-Sen University, 66#, Gongchang Road, Shenzhen, Guangdong 518107, China; Shenzhen Key Laboratory for Systems Medicine in Inflammatory Diseases, School of Medicine, Shenzhen Campus of Sun Yat-Sen University, 66#, Gongchang Road, Shenzhen, Guangdong 518107, China

## Abstract

Genetically engineered mouse models (GEMMs) are vital for elucidating gene function and disease mechanisms. An overwhelming number of GEMM lines have been generated, but endeavors to collect and organize the information of these GEMMs are seriously lagging behind. Only a few databases are developed for the information of current GEMMs, and these databases lack biological descriptions of allele compositions, which poses a challenge for nonexperts in mouse genetics to interpret the genetic information of these mice. Moreover, these databases usually do not provide information on human diseases related to the GEMM, which hinders the dissemination of the insights the GEMM provides as a human disease model. To address these issues, we developed an algorithm to annotate all the allele compositions that have been reported with Python programming and have developed the genetically modified mice information database (GMMID; http://www.gmmid.cn), a user-friendly database that integrates information on GEMMs and related diseases from various databases, including National Center for Biotechnology Information, Mouse Genome Informatics, Online Mendelian Inheritance in Man, International Mouse Phenotyping Consortium, and Jax lab. GMMID provides comprehensive genetic information on >70 055 alleles, 65 520 allele compositions, and ∼4000 diseases, along with biologically meaningful descriptions of alleles and allele combinations. Furthermore, it provides spatiotemporal visualization of anatomical tissues mentioned in these descriptions, shown alongside the allele compositions. Compared to existing mouse databases, GMMID considers the needs of researchers across different disciplines and presents obscure genetic information in an intuitive and easy-to-understand format. It facilitates users in obtaining complete genetic information more efficiently, making it an essential resource for cross-disciplinary researchers.

**Database URL**: http://www.gmmid.cn

## Introduction

The mouse is considered one of the most important model organisms in the field of biomedical research due to its many advantages, including its small size, fast reproduction, ease of breeding, advanced gene manipulation technology, and highly homologous genome compared with humans [1]. The homology between mouse and human genomes is ∼80%, and ∼99% of mouse genes have a one-to-one homolog in the human genome [2–4]. Thus, genetically engineered mouse models (GEMMs) are very useful for studying gene function in humans, providing invaluable tools in biomedical research: gene knockout and overexpression mouse models provide the most direct way to understand the physiological role of a gene in a whole animal environment; mouse models that carry the mutant alleles, which cause human genetic diseases, are irreplaceable tools for understanding the disease process and molecular mechanisms underlying the disease; GEMMs can precisely mimic human genetic diseases and thus serve as platforms for drug testing.

So far, scientists have constructed a large number of GEMMs and utilized them for various purposes in multidisciplinary research studies. It will significantly facilitate the researcher if the collective information on the GEMM involving a specific gene can be accessed in a quick and easy manner. Multiple comprehensive open-source databases have been created to provide researchers with faster and easier access to information on various genetically modified mice, such as Mouse Genome Informatics (MGI) [5], Mouse Genome Database [6], Mouse Phenome Database (MPD) [7], and Tumor Immune Syngeneic Mouse [8]. They provide information on either genetic information or phenotypic information and some other types of information, such as transcriptome data of mouse models and gene ontology analysis. However, the presentation of information in existing mouse databases is usually in highly specialized terminology, which makes it difficult to understand. For example, the genotype of a mouse model is usually presented as the form of a combination of allele symbols, lacking the annotation of each specific allele symbol or the interpretation of combining various alleles.

For researchers who are not specialized in mouse genetics, understanding the biological effect of various combinations of multiple mutant alleles can be challenging and time-consuming. Moreover, the current research trend involves fostering collaboration not only within but also beyond disciplines. As a result, many researchers who are from other fields need to quickly grasp the biological meaning of the combination of various engineered alleles in one mouse and correlated phenotypes and human disease. Current databases do not serve this goal.

Multiple systems of mouse lines have been developed to precisely control the spatiotemporal gene expression. Tissue-specific knockout is commonly accomplished using the *Cre-loxP* system [9], a versatile molecular biology tool that traces its origins back to the P1 bacteriophage [10–13]. The *Cre* recombinase, consisting of 343 amino acids, is a member of the integrase family of site-specific recombinase [14]. It recognizes a specific 34 base pair (bp) DNA sequence called *loxP* site and mediates site-specific deletion or inversion of DNA fragments between two *loxP* sites depending on the location and relative orientation of the two *loxP* sites [15–17]. DNA sequences located between two *loxP* sites placed in a cis arrangement and in the same direction will be excised by *Cre* recombinase, while the fragment between two opposingly oriented *loxP* sites will be inverted by *Cre* recombinase [18]. This system can induce the recombination of two chromosomes if *loxP* sites are placed in a trans arrangement in two chromosomes [19]. Therefore, if *Cre* recombinase is driven by a tissue-specific promoter, it will induce a genetic modification event in a tissue-specific pattern. The *CReERT2* system, an advancement of the *Cre-loxP* system, allows for more precise spatiotemporal control of genes by fusing *Cre* recombinase with the mutated ligand-binding domain of the human estrogen receptor, which results in a tamoxifen-induced *Cre* recombinase activity [20–23]. By driving *CreERT* with a tissue-specific promoter, tamoxifen treatment will introduce a genetic modification event in a tissue-specific pattern. Furthermore, the Tet-ON/Tet-OFF system, consisting of rtTA/tTA and tetracycline response element (TRE), offers flexible control over the expression of target genes, enabling researchers to precisely manipulate the initiation or cessation of gene expression in experiments [24–28]. It is also commonly utilized in mouse models to regulate gene expression [29–31]. By generating mouse lines that express rtTA, rTA, and TRE in inducible or tissue-specific patterns, the combination of these alleles in one mouse is able to precisely control spatial–temporal gene expression. However, commonly employed tools in the field of mouse genetics pose a high entry barrier: researchers frequently encounter challenges in finding relevant explanations for specialized terminology; searching the expression pattern of a tissue-specific promoter is also time-consuming, thereby augmenting the difficulty associated with reading and utilization.

For all these reasons, we have developed a mouse genetic information query system that is user-friendly for interdisciplinary researchers. We have integrated data on multiple aspects, including genetic, phenotypic, and disease-related information from existing databases, such as National Center for Biotechnology Information (NCBI) [32], MGI [33], MPD [7], Online Mendelian Inheritance in Man (OMIM) [34], and International Mouse Phenotyping Consortium (IMPC) [35], alongside with >60 000 machine-implemented and >1500 manually added annotations of various allele compositions into our system. Furthermore, we have created anatomical pictures of mouse-related tissues based on the annotation of all the mouse allele combinations. All these anatomical images were also integrated into the database. The mouse allele combination information is shown along with highlighted pictures of the anatomical parts involved, which allows readers to quickly and effectively understand the exact mouse tissues affected in the specific allele combination. In addition, we have provided links to GeneCards for information involving human homologous genes and diseases [36]. The genetically modified mice information database (GMMID) will help researchers across multidisciplines to quickly grasp the genetic events of each GEMM and connect the murine models with phenotypes and human diseases, allowing quick understanding of the physiological role of genes and facilitating subsequent experiment design. GMMID is freely available at http://www.gmmid.cn.

## Materials and methods

### Data collection

In order to create a comprehensive and high-standard gene dataset, we implemented a data collection process consisting of three main steps. First, all the murine genes from the website of the National Institutes of Health (NIH) (https://www.ncbi.nlm.nih.gov/gene/) were collected, and gene ID, gene symbol, and alias were collected as well. Second, the human homolog of each gene was collected from the MGI database (http://www.informatics.jax.org/). All the existing alleles of a specific gene, including spontaneous mutations, chemical-induced mutations, targeted modifications, and transgenic modifications, were gathered from the MGI database. Moreover, GEMMs that have been reported, phenotypes, and references of the GEMM were also collected from the MGI database. Furthermore, we have acquired mouse phenotypic data associated with mouse genotypes from the IMPC (https://www.mousephenotype.org/). Finally, the related diseases of human homolog of the specific gene were gathered from the OMIM website (https://www.omim.org/). Biological descriptions on compositions of alleles in each GEMM were generated as described in the following section. Anatomical diagrams of mouse tissues mentioned in the biological descriptions of every GEMM were also created. Overall, we collected information on 80 646 genes and 65 106 allele compositions that met our requirements and put them into eight tables. The information of registered ID and password is also stored in a table named “users.” The relations among the information are stored in eight relation tables. In all, there are 10 entity tables and eight relation tables consisting of the database of GMMID (Table 1).

**Table 1.** Tables in GMMID

Type	Table name	Content
Entity table	Gene	Gene ID, gene symbol, gene alias, human homolog, GeneCardsurl, IMPCurl
	Allele composition	Data on allele composition of GEMMs
	Double alleles	Two alleles of the same gene
	Gene allele	Modified allele and the type of modification
	Disease	Causal mutation, disease description, phenotype
	Phenotype	Description of biological change
	Reference	References of GEMMs
	Tissues	Tissues of all the anatomical images
	Users	Registered ID and password
Relation table	Gene_allele composition	Relation between gene and allele composition
	Allele composition_double alleles	Relation between allele composition and double alleles
	Double alleles_gene allele	Relation between double alleles and gene allele
	Gene category_disease	Relation between gene category and disease
	Allele composition_phenotype	Relation between allele composition and phenotype
	Allele composition_reference	Relation between allele composition and reference
	Gene category_reference	Relation between gene category and reference
	Allele composition_tissues	Relation between allele composition and tissues

### Algorithm of allele composition annotation

**Figure 1.**
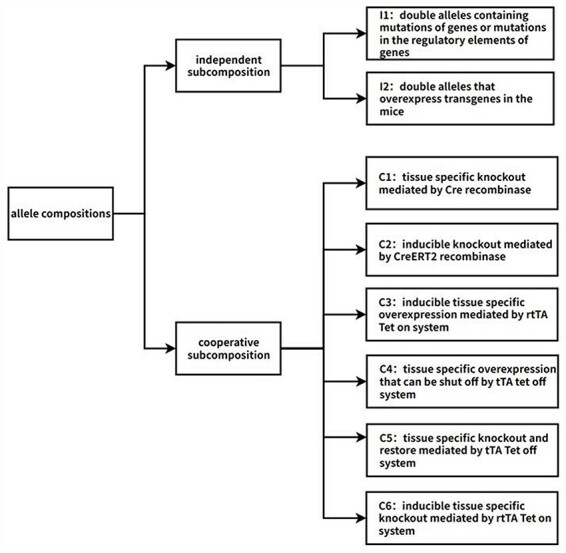
Flow chart of the algorithm of adding biological descriptions to allele compositions. The first step is to split allele compositions into two types of subcompositions: independent subcomposition and cooperative subcomposition. Independent subcompositions are the double alleles that act independently and can be categorized into two types: I1, double alleles containing mutations of genes or mutations in the regulatory elements of genes; I2, double alleles that overexpress transgenes in the mice. Cooperative subcompositions can be categorized into six types, namely C1: tissue-specific knockout mediated by *Cre* recombinase; C2: inducible knockout mediated by *CreERT2* recombinase; C3: inducible tissue-specific overexpression mediated by the rtTA Tet-on system; C4: tissue-specific overexpression that can be shut off by the tTA Tet-off system; C5: tissue-specific knockout and restore mediated by the tTA Tet-off system; C6: inducible tissue-specific knockout mediated by the rtTA Tet-on system. Automated annotation is performed based on these categories. If some allele compositions do not belong to any category, they are screened out for manual annotation. So far, there are >60 000 machine-annotated allele compositions and >1500 manually annotated allele compositions.

Information of all allele compositions was collected from the MGI database. Every allele composition corresponds to one GEMM (Fig. 1). To annotate the allele compositions, they were first analyzed and subdivided into subcompositions, which represent individual biological events involving one target gene. Two types of subcompositions were identified: independent and cooperative. The independent subcomposition consists of the independent double alleles, which typically consist of mutation or transgene alleles. The cooperative subcomposition consists of multiple double alleles that work together to produce a single biological event. Each subcomposition within the allele composition is annotated, and the final annotation for the allele composition is a combination of all subcomposition annotations.

The independent alleles represent biological events on their own. They can be subdivided into two categories: I1. Double alleles containing mutations of genes or mutations in the regulatory elements of genes. The mutations are usually chemically induced mutations, radiation-induced mutations, gene trap–generated mutations, or targeted mutations generated by gene editing techniques. These double alleles are straightforward to understand and were annotated by the way how the mutations are generated. Consider the *Zfp541<em1Osb>/Zfp541<em1Osb>* as an example. The allele *Zfp541<em1Osb>* is a null allele generated by the *Crispr-Cas9* system, so it is annotated as “Zfp541 conventional knockout (global knockout).” I2. Double alleles that overexpress transgenes in the mice. These transgenes are usually overexpressed ubiquitously or in specific tissues depending on the promoters that drive the transgenes. We annotated the expression pattern based on the information of the promoters, which is collected from the MGI website [37]. For example, *Tg(Myh6-Acsl1)J3Jesc/0* is a typical I2 subcomposition, *Myh6* drives expression in the cardiovascular system, and thus this subcomposition is annotated as “Overexpressing Acsl1 in cardiovascular system.”

The cooperative subcompositions consist of multiple alleles that cooperate with other alleles to exert functions. These combinations of multiple double alleles are usually hard for interdisciplinary scientists to understand. The cooperative subcompositions can be subdivided into six categories for further analysis and annotation. The six categories are defined and annotated as follows: C1. Tissue-specific knockout mediated by *Cre* recombinase. These subcompositions contain alleles of tissue-specific *Cre* recombinase and floxed alleles. The floxed allele is specifically inactivated in the tissue where the *Cre* recombinase is expressed. The expression pattern of *Cre* recombinase is determined by the promoter that drives the *Cre* recombinase. The promoter activity is annotated based on the information of the MGI website [37]. For example, the allele *Lepr<tm1.1Chua>* is a floxed allele, and the *Agrp<tm1(cre)Lowl* allele is an allele that drives neuron-specific Cre recombinase expression. So, the subcomposition “*Agrp<tm1(cre)Lowl>/Agrp<+> Lepr<tm1.1Chua>/Lepr<tm1.1Chua>*” is annotated as “Knockout Lepr specifically in neurons”. C2. Inducible knockout mediated by *CreERT2* recombinase. These subcompositions contain alleles of tissue-specific *Cre* recombinase fused to a mutant estrogen ligand-binding domain (*ERT2*) and floxed alleles. The *Cre* recombinase expression requires the presence of tamoxifen. The expression pattern of *Cre* recombinase is determined following the procedure in the C1 category. For example, the *Gt(ROSA)26Sor<tm1(cre/ERT2)Thl>* allele drives *CreERT2* expression ubiquitously, and *Mcl1<tm1Ywh>* is a floxed allele. So, the subcomposition *Gt(ROSA)26Sor<tm1(cre/ERT2)Thl>/Gt(ROSA)26Sor<+> Mcl1<tm1Ywh>/Mcl1<tm1Ywh>* is annotated as “Knockout Mcl1 ubiquitously in a tamoxifen-inducible manner.” C3. Inducible tissue-specific overexpression mediated by the rtTA Tet-on system. These subcompositions contain tissue-specific rtTA alleles and alleles of genes driven by tet-O (tetracycline operator sequence). Administration of tetracycline/doxycycline will initiate the expression of the genes driven by tet-O in the specific tissues where rtTA is expressed. The expression pattern of rtTA is determined by the promoter that drives the rtTA. The promoter activity is annotated based on the information of the MGI website [37]. The subcomposition “*Tg(Scgb1a1-rtTA)1Jaw/0 Tg(tetO-Aimp1)29872Mcla/0*” is an example of this category. *Scgb1a1* drives the rtTA expression in the respiratory system, so this allele subcomposition is annotated as “tetracycline/doxycycline treatment induced overexpression of *Aimp1* in respiratory system.” C4. Tissue-specific overexpression that can be shut off by the tTA tet-off system. These subcompositions contain tissue-specific tTA alleles and alleles of genes driven by tet-O. Genes driven by tet-O are overexpressed in the specific tissue where tTA is expressed. Administration of tetracycline/doxycycline will shut off the overexpression. The expression pattern of tTA is determined by the promoter that drives the tTA. The promoter activity is annotated based on the information of the MGI website [37]. C5. Tissue-specific knockout and restore mediated by the tTA Tet-off system. These subcompositions contain alleles of *Cre* recombinase driven by tet-O, tissue-specific tTA allele, and floxed alleles. These subcompositions generate tissue-specific knockout of the floxed alleles, while the Cre recombinase expression can be shut off by tetracycline/doxycycline treatment. The tissue of knockout is determined by the tissue of the tTA allele, which is annotated following the procedure in C4. Once the *Cre* recombinase expression is shut off, tissue-specific knockout will not be induced. The expression of the floxed gene will be restored once new cells were generated from stem cells that were not excised previously. For example, in this allele subcomposition “*Hnf4a<tm1Sad>/Hnf4a<tm1Sad> Six2<tm1(tTA*,*tetO-EGFP/cre)Amc>/Six2<+>*,” *Six2* drives the tTA expression in renal and urinary system, especially in metanephric mesenchyme. *Cre* and *EGFP* are both driven by tetO, so the *Cre* recombinase and *EGFP* are coexpressed. As a result, the *EGFP* marks the expression pattern of *Cre* recombinase. This subcomposition is annotated as “Inactivating Hnf4a in renal & urinary system, especially in metanephric mesenchyme. Inactivation is marked by EGFP, tetracycline/doxycycline treatment restore the expression of Hnf4a.” C6. Inducible tissue-specific knockout mediated by the rtTA Tet-on system. These subcompositions contain alleles of the *Cre* recombinase driven by tet-O, tissue-specific rtTA allele, and floxed alleles. The *Cre* recombinase is expressed upon tetracycline/doxycycline treatment, which induces excision of the floxed alleles. The expression site of *Cre* is determined by the tissue of the rtTA allele, which is annotated following the procedure in C3. For instance, in the allele combination “Pkd1<tm2Ggg>/Pkd1<+> Tg(Pax8-rtTA2S*M2)1Koes/0 Tg(tetO-cre)1Jaw/0,” the *Pax8-rtTA* construct incorporates an optimized rtTA variant *(rtTA2s-M2)* cDNA and *SV40* polyA sequence, replacing the endogenous ATG translational start site of a murine Pax8 sequence, which drives the expression of rtTA in the kidney. Therefore, it is annotated as “Inactivating Pkd1 heterozygously in the kidney. The inactivation is induced by tetracycline(or doxycycline) treatment.”

### Website development

In order to provide the public with a more systematic and convenient way to access information on genetically modified mice, we have developed a user-friendly website system that allows users to search and display GEMM data. The website, which is accessible at http://www.gmmid.cn, is implemented using the SpringBoot framework and deployed on a CentOS cloud server. It is comprised of five functional modules: Home, Login/Register, Search, Data Display, and Backend Management.

The Home page is the first page that users see upon entering the website. From there, users can choose to register, log in, or search for information. The Login/Register module allows users to register and log in to record their gene query history, which can be beneficial for future data analysis. It is important to note that registration and login are optional, and users can still access all website functions if they choose not to register or log in.

The Search module is divided into five submodules, each focusing on a specific search direction: gene category, allele combination, allele pair, allele, and phenotype. Each submodule presents search results and detailed information related to its search category. Within the phenotype search submodule, we have implemented a fuzzy search based on querying keyword cosine similarity calculation. When users search for phenotype information related to gene-modified mice, the system provides real-time recommended semantic similar results, with the top 10 being displayed. This allows for efficient and accurate data search, even if users have not entered complete search keywords.

Moreover, each submodule is interrelated and linked based on data relationships. This allows users not only to obtain detailed information about their query objects but also to more comprehensively understand the relationships between genes, phenotypes, diseases, and other information.

In the backend management module, website administrators can statistically analyze the most commonly searched data based on the “search terms” collected during login and registration. During later website maintenance stages, administrators will update, supplement, and improve the gene information, biological description information, and mouse tissue anatomy diagram of GEMM based on these statistics. Additionally, administrators can manage existing gene-modified mouse data, update new data in real time, modify incorrect data, and delete old data.

## Results

### Genetic data statistics

**Figure 2.**
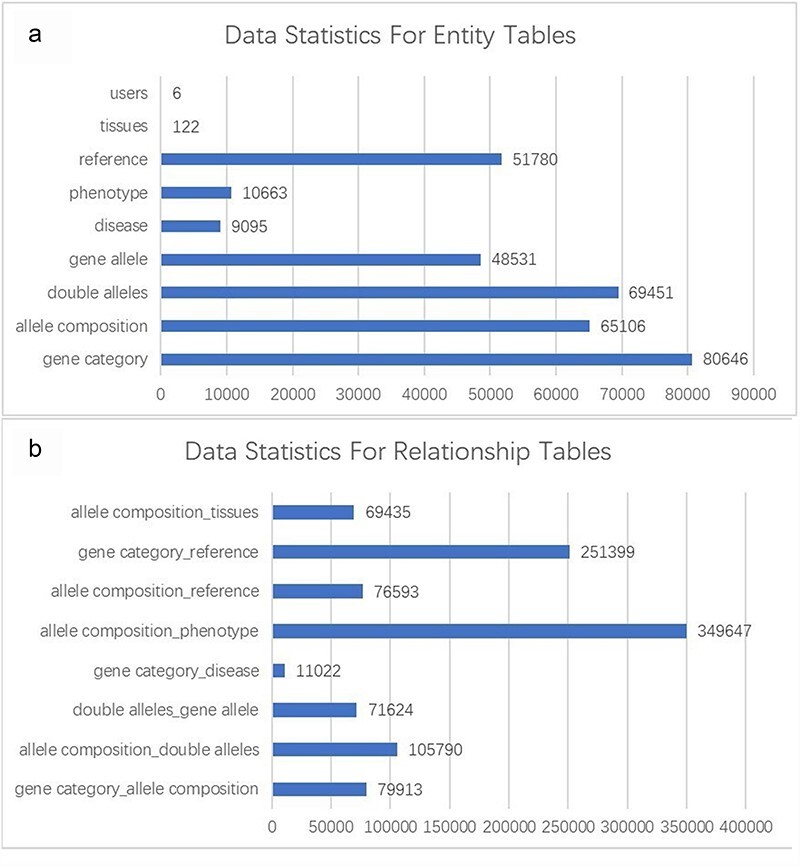
Statistical analysis of entity and relationship tables in the GMMID database. (a) Statistical analysis of entity table data. (b) Statistical analysis of relationship table data.

The GMMID database currently comprises a total of 80 646 murine gene records, 65 106 allele composition records, 69 451 double allele records, 48 531 gene allele records, 10 663 phenotype records, and 51 780 related literature records. The gene records include information on gene ID, gene symbol, and alias, which were collected from the website of the NIH (https://www.ncbi.nlm.nih.gov/gene/). Human disease relevant to the gene provides important information for understanding the role of the gene; however, the existing database usually only provides information on mouse and a website link to OMIM, which prevents the researchers from quickly getting a whole picture of the gene. Therefore, the brief information of relevant human diseases and website links is provided on GMMID. Of human diseases, 9095 records were collected from the OMIM website (https://www.omim.org/) based on the human homolog gene collected from the MGI database (http://www.informatics.jax.org/). Additionally, we obtained 80 646 mouse phenotype data related to mouse genotypes from the IMPC. Moreover, the records of mouse models with a combination of different alleles that have been reported were collected from MGI as well. We have conducted a statistical analysis of the database (Fig. 2a and b). Data cleaning was done by Python to remove incorrect or duplicated data.

### Biological description on allele composition of GEMMs

Existing databases on mouse models usually describe alleles in detail, including the specific technique and procedure used to generate the allele, the genetic background and the detailed information on the manipulation of sequence, the phenotype of mouse that carries this specific allele, and the potential usage of this allele. However, these are not informative for cross-disciplinary researchers who need to understand the physiological role of a specific gene quickly. It is time-consuming for researchers to figure out the biological effect of a combination of different alleles. GMMID is aimed to solve this problem. To this end, the biological effects of all combinations of different alleles are annotated in GMMID.

**Figure 3.**
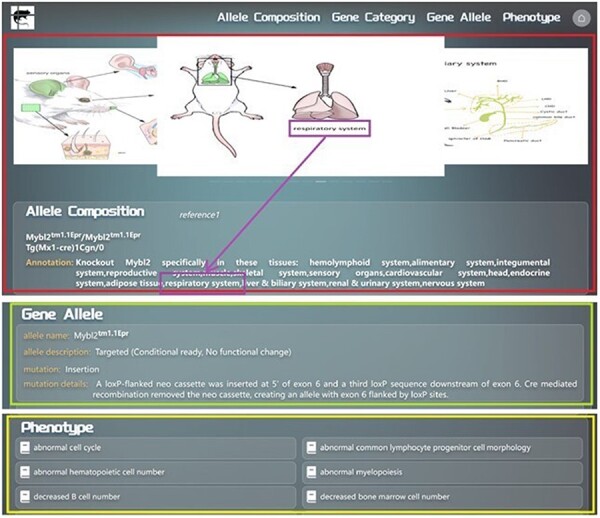
Detailed layout description of the information display page for searching or navigating to the allele composition *Mybl2tm1.1Epr/Mybl2tm1.1Epr Tg(Mx1-cre)1Cgn/0* in the GMMID database. Redbox: self-information section of the allele composition on the information display page. Green box: gene allele information section on the information display page. Yellow box: phenotype information section on the information display page. Purple box: anatomical images of genetically modified mice corresponding to the mentioned tissues in the annotation. Blue box: links to references associated with this allele composition.

We developed an algorithm to annotate all the allele compositions by machine programming. For those allele compositions, which are too complicated to be annotated by this algorithm, manual annotations are added. Annotations of every allele composition are presented alongside the allele composition. We will use the example of searching for *Mybl2^tm1.1Epr^/Mybl2^tm1.1Epr^ Tg(Mx1-cre)1Cgn/0* to provide a detailed overview of the allele composition information display page in GMMID. The allele composition information display page consists of three main sections. First, there is the self-information of the allele composition (Fig. 3), which includes the alleles of the composition, annotation of the composition, and relevant mouse anatomical images illustrating the tissues or organs mentioned in the annotation (Fig. 3). Secondly, the information of each allele in this composition is listed (Fig. 3). Lastly, phenotypes related to the allele composition are listed on this page as well (Fig. 3). The links to references for all the information on the current page are listed in the self-information area (Fig. 3), allowing users to conveniently access detailed information.

### Processing of mouse genetic information search

**Figure 4.**
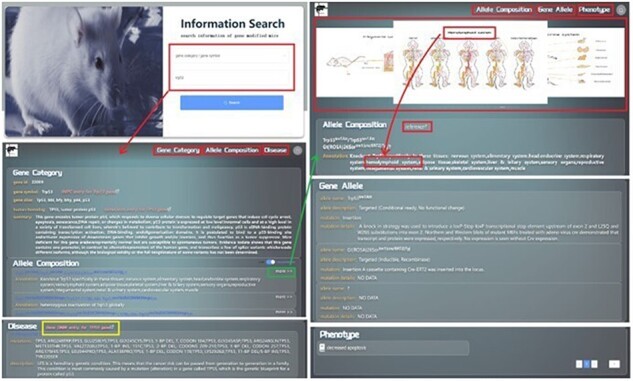
Presentation Diagram of User Information Search Process on GMMID. The web pages when searching with the gene symbol “*Trp53*.” Initially, the website presents the relevant information page for the “*trp53*” gene category, which includes details about the gene ca itself, related allele composition information, and pertinent human homologous disease information. The yellow box is the hyperlink to the OMIM gene entry. Upon clicking on a specific allele composition, the page will redirect to a display page showcasing comprehensive information about that allele composition. This includes its own details, annotated mouse anatomical images, relevant reference links, gene allele information, and associated phenotype information, among other relevant data.

GMMID provides a user-friendly interface for browsing and querying information on genetically modified mice. The homepage of GMMID features an Information Search module where users can select from five categories, including gene category, allele composition, double alleles, gene allele, and phenotype, to query gene information on genetically modified mice. For instance, if a user chooses to search for the gene *Trp53* by using its gene symbol in the search field on the homepage, the interface will automatically redirect to the detailed page of the *Trp53* gene (Fig. 4a). In the Log In | Register module, users can register or log in to their account for backend recording and analysis of their search information. This information provides a basis for the improvement of GMMID in the future. It is worth noting that registration and login are optional and do not restrict guest users from browsing or querying information.

**Figure 5.**
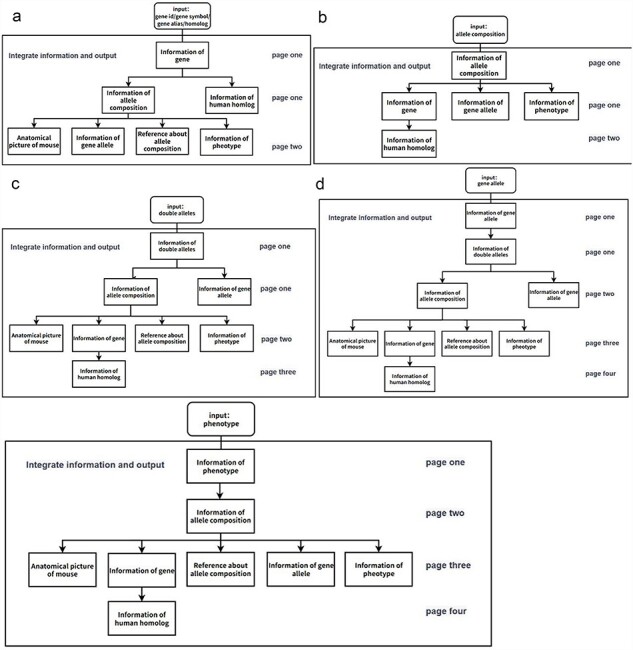
Flowchart of the information display of the page when a user searches for mouse information in the GMMID database interactive interface. (a) Flowchart of the information display of the page when a user searches for a specific gene in the GMMID database interactive interface. On the first page, the displayed information includes self-information on the gene category, information about allele composition related to the gene category, and relevant information on human homologous diseases. On the second page, specific self-information of a particular allele composition is presented, along with mouse anatomical images mentioned in the annotations, relevant gene allele information, related literature information, and corresponding phenotype information. (b) Flowchart of the information display of the page when a user searches for a specific allele composition in the GMMID database interactive interface. (c) Flowchart of the information display of the page when a user searches for specific double alleles in the GMMID database interactive interface. (d) Flowchart of the information display of the page when a user searches for a specific gene allele in the GMMID database interactive interface. (e) Flowchart of the information display of the page when a user searches for a specific phenotype in the GMMID database interactive interface.

On the gene information page, there are five different information display pages corresponding to the five different search methods (Fig. 5a–e). For example, when users select “gene category” to query gene-related information on genetically modified mice, the page displays specific details of the gene, including gene ID, gene symbol, gene alias, human homolog, and all the GEMMs (allele composition of each GEMM is shown). Additionally, the page displays information related to diseases associated with the human homolog gene. Genetic mutation and a brief description of the disease are shown on this page, and users can also quickly access the relevant pages in OMIM for more information from this page. Users can also click on the allele composition to view. The relevant allele composition information page displays the biological description of the allele composition and related mouse anatomy diagram. The page also displays phenotype and reference information related to the allele composition to enable users to obtain a comprehensive understanding of the genetically modified mice quickly and clearly (Fig. 4).

## Discussion

Genetically modified mice are a crucial animal model for clinical trials, and there are now tens of thousands of gene modifications available for them. However, those data are mostly scattered across different databases, lacking clear and concise biological descriptions to aid interpretation. As a result, data queries become less efficient, and understanding the information becomes more challenging, especially for interdisciplinary scientists. GMMID is the first database to address and solve these issues by providing a complete data chain that integrates information from several professional databases for mice and rats. It also caters to nonprofessional users’ search and reading needs by adding biological descriptions and anatomical images to help them understand the gene information. The user-friendly and straightforward interactive page further enhances users’ ability to efficiently obtain relevant information on genetically modified mice. GMMID will help users across different fields to grasp and obtain genetically modified mouse-related information efficiently and contribute to the research involving mouse models greatly.

Currently, there is still room for the improvement of GMMID. For instance, the hand-drawn mouse tissue anatomy images presented on the allele composition page lack details. In forthcoming maintenance updates, we aim to furnish more refined anatomical illustrations. Moreover, we aim to incorporate the publicly available multi-omics data of each gene into GMMID in the future updates.

## Conclusion

We have created a user-friendly mouse genetic information query system that integrates genetic, phenotypic, and disease-related data from various databases, including NCBI, MGI, MPD, OMIM, and IMPC. The system includes >60 000 machine-implemented and >1500 manually added annotations of allele compositions. Additionally, we have incorporated anatomical pictures of mouse-related tissues into the database to help researchers quickly understand the specific tissues affected. This system, called GMMID, facilitates the understanding of genetic events in GEMMs, connects murine models with phenotypes and human diseases, and is freely available at http://www.gmmid.cn.

## Data Availability

All data used in this study are publicly available at http://www.gmmid.cn.
